# Role of Epigenetic Regulation in Plasticity of Tumor Immune Microenvironment

**DOI:** 10.3389/fimmu.2021.640369

**Published:** 2021-04-02

**Authors:** Yunkai Yang, Yan Wang

**Affiliations:** State Key Laboratory of Molecular Oncology, National Cancer Center, National Clinical Research Center for Cancer, Cancer Hospital, Chinese Academy of Medical Sciences and Peking Union Medical College, Beijing, China

**Keywords:** DNA methylation, histone modification, ncRNAs, TIME, ITH, epigenetics

## Abstract

The tumor immune microenvironment (TIME), an immunosuppressive niche, plays a pivotal role in contributing to the development, progression, and immune escape of various types of cancer. Compelling evidence highlights the feasibility of cancer therapy targeting the plasticity of TIME as a strategy to retrain the immunosuppressive immune cells, including innate immune cells and T cells. Epigenetic alterations, such as DNA methylation, histone post-translational modifications, and noncoding RNA-mediated regulation, regulate the expression of many human genes and have been reported to be accurate in the reprogramming of TIME according to vast majority of published results. Recently, mounting evidence has shown that the gut microbiome can also influence the colorectal cancer and even extraintestinal tumors *via* metabolites or microbiota-derived molecules. A tumor is a kind of heterogeneous disease with specificity in time and space, which is not only dependent on genetic regulation, but also regulated by epigenetics. This review summarizes the reprogramming of immune cells by epigenetic modifications in TIME and surveys the recent progress in epigenetic-based cancer clinical therapeutic approaches. We also discuss the ongoing studies and future areas of research that benefits to cancer eradication.

## Introduction

Cancer is the leading cause of death worldwide and in China and thus remains as the single biggest stumbling block for extending life expectancy. According to GLOBCAN 2018, there are approximately 18.1 million new cancer cases and 9.6 million new cancer deaths worldwide, 24 and 30% of which occur in China, respectively (1, 2). This suggests a large gap between China and other developed countries, such as the United States, in terms of cancer mortality (1, 3). Thus, new insights into cancer therapy are necessary for the development of novel strategies and efficacious drug combination therapies.

Tumors are not only a group of abnormally proliferative cells, but also a special environment termed as the tumor microenvironment (TME) that contains different cell types, including tumor and immune cells (4). Owing to the large number of immunosuppressive immune cells, the TME is also called TIME. Thus, developing therapeutic approaches targeting the plasticity of TIME has become one of the most attractive area in cancer therapy. Immune checkpoint inhibition (ICI) is a promising strategy that involves the activation of the function of TIME T cells to combat tumor cells (5, 6). However, the majority of cancer patients exhibited minimal or no clinical response to ICI therapy (5).

Epigenetic changes in genes encoding tumor suppressors, inhibitory cytokines, and immune checkpoint molecules, e.g., PD-L1 and CD47, can lead to impaired anti-cancer immunity, uncontrollable tumor growth, immune escape, and drug resistance, eventually resulting in tumor development, progression, and metastasis (7, 8). Therefore, targeting the epigenetic alterations in cancer cells with epigenetic-associated drugs (epi-drugs) could convert a tumor from an immune suppressive (cold) to an immune permissive (hot) state (9). This could improve the therapeutic effects of other anti-tumor drugs, especially immune checkpoint inhibitors (ICIs). Within the TIME, epigenetic modifications can also be found in tumor-associated immune cells, including myeloid cells, CD4^+^ T cells, and CD8^+^ T cells (9–11). During the differentiation from naïve CD8^+^ T cells to CD8^+^ effector T cells, epigenetic changes, such as DNA methylation and histone modifications, are involved in the chromatin accessibility (12, 13). The immune checkpoint protein PD-1 expressed on the surface of exhausted T cells is also regulated by DNA methylation (14). Thus, disrupting the unusual epigenetic regulation in cancer can completely shape the TIME by decreasing the populations of immunosuppressive cells, such as tumor-associated macrophages (TAMs) and myeloid-derived suppressor cells (MDSCs) (15), increasing the numbers of CD8^+^ effector T cells and NK cells (15, 16), elevating the levels of inflammatory cytokines and chemokines (17–19), and upregulating the expression of tumor antigens, such as cancer/testis antigens (CTAs) (20, 21).

Tumor heterogeneity, especially intratumor heterogeneity (ITH), is one of the major hallmarks of cancer. Within TIME, there is diversity in the phenotypes of tumor cells and the infiltration and differentiation status of immune cells, and the diversity is characterized by distinct microscopy fields of a single biopsy. Tumor or TIME is formed from a single mutated cell that abnormally proliferates and accumulates additional mutations through Darwinian evolution (22). This may cause drug resistance to cancer therapy, such as in patients with breast cancer, due to pre-existing resistant subclones within the tumor verified by single-cell sequencing technique (23). Aberrant epigenetic changes occur more frequently than gene mutations in human cancer. Thus, targeting the epigenetic changes in cancer may reverse drug resistance to cancer therapies, particularly immunotherapies, and increase the efficacy of other therapeutic approaches that initially failed to achieve durable responses, which is always attributed to ITH (24).

In this review, we summarize the recent knowledge on the role of epigenetic modifications in TIME and ITH. In addition, the latest clinical therapeutic approaches are discussed. These epigenetic alterations may serve as potential targets for more efficacious therapeutic intervention in cancer.

## Epigenetic Modifications

**Figure 1:**
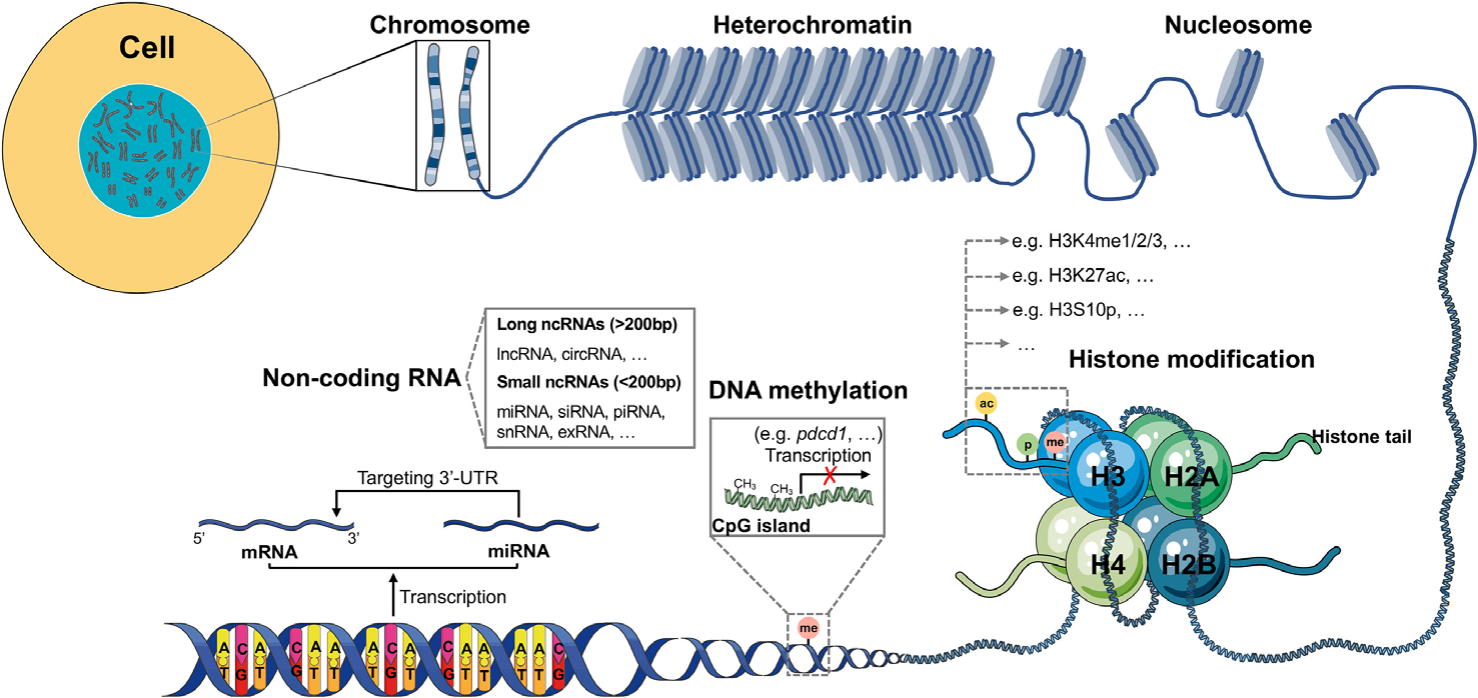
Schematic model of epigenetic regulation. The expression of most human genes is regulated by epigenetic modifications. There are three different epigenetic processes that control gene transcription and expression: DNA methylation, histone modification and ncRNA. DNA methylation always exists in GC-rich areas of the human genome called CpG islands, which can be methylated by DNMTs, resulting in failed transcription of genes, such as *pdcd1*. Histone modification, in which amino acids on four different histone tails (H2A, H2B, H3, H4) can be modified by different enzymes (KMTs, HATs, phosphatases, KDMs, HDACs, among others), results in the regulation of gene expression. In the human genome, many DNA sequences cannot be transcribed into mRNAs but are transcribed as ncRNAs. According to the length, ncRNAs can be divided into small and long ncRNAs. The most investigated ncRNA is miRNA, which targets the 3’-UTR of mRNA, thus contributing to gene silencing.

Epigenetics refers to a special cell events causing heritable phenotypic changes but do not involve alterations in the DNA sequence. Epigenetic modifications involve three different processes, namely DNA methylation, histone modifications, and non-coding RNAs (ncRNAs). They are critical in the regulation of the aberrant expression of tumor-associated genes and encoding of immune checkpoint proteins, tumor suppressors, or oncoproteins in cancer, that contribute to tumor progression and immune invasion (**Figure 1**). Hence, targeting the dysregulation and dynamic nature of epigenetic alterations provides a new strategy for cancer therapy.

### DNA Methylation

DNA methylation is a biological process in which methyl groups (–CH_3_) from S-adenosylmethionine (SAM) are added to the 5’ position of the pyrimidine ring of cytosines in the CpG dinucleotide called CpG island. Adenine methylation has been recently observed in mammalian DNA (25), although it has attracted less attention. Gene transcription is silenced when CpG-rich promoters are hypermethylated as these methylated CpGs can impair the binding of transcriptional factors and recruit repressive complexes (26). DNA methylation always represses the expression of tumor-suppressive genes in many types of cancer (27). The process of DNA methylation is mediated by DNA methyltransferases (DNMTs), which include DNMT1, DNMT3a, and DNMT3b (28, 29). DNMT2, a homolog of DNMTs, contains all 10 motifs common to all DNMTs. However, DNMT2 can methylate cytosine-38 in the anticodon loop of aspartic acid transfer RNA (tRNAAsp), instead of DNA (30). In gene promoters, DNA methylation occurs in correlation with gene silencing, whereas in other regions, it modulates enhancer activity, gene activation, and splicing (31, 32). For example, in the promoter region of the *pdcd1* gene, more methylated sites were observed in PD-1^low^ A20 cells than in PD1^high^ EL4 cells, indicating that DNA methylation occurring in the promoter region silences the expression of PD-1 in T cells (14).

5-methylcytosine (5mC) can be removed *via* oxidation catalyzed by ten-eleven translocation (TET) methylcytosine dioxygenases (TET1, TET2, and TET3), resulting in generation of 5-hydroxymethylcytosine (5hmC), 5-carboxycytosine (5caC), 5-formylcytosine (5fmC), and unmethylated cytosine (33, 34). DNMTs and TETs regulate the gene activation and repression, together maintaining the stability of gene transcription under certain circumstances. Once this balance is interrupted, many genes are abnormally silenced or activated, leading to various pathological conditions, especially cancer (35). In patients with primary breast cancer (PBC) and colorectal cancer (CRC), immune checkpoint proteins PD-L1, CTLA-4, TIGIT and TIM-3 are significantly upregulated with the hypomethylation of promoters because of upregulated TET2 and TET3 (36). The increased levels of immune checkpoint molecules may be one of the causes of repressed activation and function of immune cells in the TIME.

In a pan-cancer analysis result, researchers found that the global loss of DNA methylation is negatively correlated with host immune pathways, including antigen processing and presentation, cytokine–cytokine receptor interaction, and major histocompatibility complex (MHC) (37). In the same study, DNA demethylation has a positive correlation with genomic mutation burden and aneuploidy level, which contributes to tumor cell proliferation (37). Therefore, DNA methylation-modifying agents can be potentially used for cancer therapy or the improvement of the efficacy of cancer immunotherapy. DNA methylation also acts as a modulator of immune cells differentiation. Datasets from the BLUEPRINT Epigenome Project (http://www.blueprint-epigenome,eu) reveal that the global methylation level increases during macrophage differentiation and activation, whereas it acts in an opposite way in T and B cells (38).

### Histone Modifications

There are two types of histones: core histones H2A, H2B, H3, H4 and linker histone H1. They can be modified by proteins called “readers,” “writers,” and “erasers” at the histone tails. The nucleosome core comprises two H2A–H2B dimers and an H3–H4 tetramer. The most frequent histone modifications are methylation, acetylation, and phosphorylation; however, there exist other modifications, including citrullination, ubiquitination, ADP-ribosylation, deamination, formylation, O-GlcNAcylation, propionylation, butyrylation, crotonylation, proline isomerization, and lactylation (39–41). All of these modifications not only activate or repress gene transcription, but also influence several processes, such as DNA repair, DNA replication, and recombination (40). Once histone modifications are aberrantly regulated, the steady state of the cell is disrupted, and diseases, such as cancer initiate, develop, and progress.

#### Histone Methylation

Unlike DNA methylation, histone methylation involves the addition of methyl groups to mainly lysine (K) (mono-, di-, or trimethylated) and arginine (R) residues (mono- or dimethylated) in the histone tails, which mediate gene transcription, including those cancer progressive and immunosuppressive genes. The six major families of histone lysine methyltransferase complexes (KMT1-6) are responsible for the methylation of lysine residues, mainly on histone H3, followed by H4 (42, 43). The methyl groups added to lysine residues by KMTs can be removed by lysine demethylases (KDMs), which contains six families (KDM1-6) at least (8). The distinct sites or degrees of lysine methylation on histones determine the activation or silencing of many genes. For instance, methylation at lysine 4 on histone H3 (H3K4me1/2/3) and H3K36me2/3 are always involved in the activation of gene transcription, whereas that on H3K9me3 and H3K27me3 exert the opposite function (8, 44). The loss of H3K79me2 in TIME contributes to tumor progression in a mouse model (45). Many immune cell types, such as macrophages, dendritic cells (DCs), and natural killer cells (NKs), can also be regulated by histone methylation in cancer (46–48).

#### Histone Acetylation

Histone acetylation is involved in the activation of gene transcription by attenuating interactions between histones and DNA *via* the addition of an acetyl group (–CH_3_CO) from the acetyl coenzyme A (acetyl-CoA) to the α/ϵ-amino group of lysine side chains, as it neutralizes the positive charge (40, 41, 49, 50). The reversible addition and removal of acetyl groups are catalyzed by histone acetyltransferases (HATs) and histone deacetylases (HDACs) (51). There are two types of HATs (type-A and type-B) found in the human genome, of which, type-B HATs can only acetylate newly synthesized histones, such as H4 at K5 and K12, but not those deposited in the chromatin (40, 52). The well-studied and major families of HATs in humans include GNAT (HAT1, GCN5, and PCAF), MYST (Tip60, MOF, MOZ, MORF, and HBO1), and p300/CBP (53). On the other hand, the loose chromatin mediated by HATs can be restabilized by HDACs, resulting in transcriptional silencing. HDAC1, a component of the NuRD complex, mediates the histone deacetylation of H3K27 in the promoter region of STAT1, which downregulates STAT1 expression, resulting in type I IFN suppression in TIME (54). HDACs can be classified into four groups (I, II, II, and IV) (53). HDACs, as potential cancer therapeutic targets, have attracted increasing attention due to their role in cancer epigenetics and disease development. Currently, there are four FDA-approved HDAC inhibitors: Vorinostat (SAHA) and Istodax (romidepsin) have been approved for the treatment of cutaneous T-cell lymphoma (CTCL) in 2006 and 2009, respectively; Beleodap has been approved for the treatment of peripheral T-cell lymphomas (PTCL) in 2014; and Panobinostat has been approved for the treatment of patients with multiple myeloma (MM) in 2015 (55). HDAC inhibitors have multiple functions in immunomodulatory activities, including the promotion of the expression of MHC I molecule, tumor antigens, PD-L1, and T cell chemokines, induction of immunogenic cell death hallmarks in tumor cells, and decreasing Treg cells (13, 56, 57). Metabolites, such as butyrate and propionate, produced by the gut microbiome can also inhibit the activity of HDACs (58).

#### Histone Phosphorylation

Histone phosphorylation, another post-transcriptional modification (PTM) event, occurs mainly at the serine (S), threonine (T), and tyrosine (Y) sites of histone tails and regulates the transcription of genes that are involved in cell cycle and proliferation (27, 59). Histone phosphorylation is correlated with the proliferation and progression of many types of cancer. For instance, decreased H3S10p levels were observed in MDA-MB-231 cells treated with the microRNA-941 inhibitor, which suggests that H3S10p has a potential role in promoting the proliferation of MDA-MB-231 cells (60). The tyrosine 39 of histone H2A.X can be phosphorylated by JMJD6, which leads to triple-negative breast cancer (TNBC) cell growth (61). In castration-resistant prostate cancer (CRPC), researchers have found that histone phosphorylation is positively correlated with cancer cells progression and drug resistance, and its blockade inhibits tumor growth in a CRPC mouse model (62).

### Non-Coding RNAs

RNAs that are not translated into proteins are termed as ncRNAs, which represent about 90% of human genome-derived RNAs and contain small ncRNAs, such as microRNAs (miRNAs), small interfering RNAs (siRNAs), PIWI-interacting RNAs (piRNAs), small nuclear RNAs (snRNAs), extracellular RNAs (exRNAs), circular RNAs (circRNAs), and long non-coding RNAs (lncRNAs), such as Xist (27, 63). Small ncRNAs are less than 200 bp in length, whereas circRNAs and lncRNAs are more than 200 bp in length (27). The aberrant expression of ncRNAs is always associated with many diseases, including cancer. One of the most widely studied ncRNAs is miRNAs, which are nearly 20 bases long and mediate the cleavage and degradation of mRNAs by targeting the 3’-untranslated region (3’-UTR), thereby leading to translation failure (64).

Thousands of miRNAs have been found to regulate >30% of human genes engaged in the cell cycle, and cell proliferation, differentiation, or apoptosis (65–67). Some miRNAs can act as tumor suppressors by targeting immune checkpoint molecules, such as PD-L1, PD-1, CTLA-4, and TIM-3, in tumor cells, such as ovarian cancer, prostate cancer (PC), and non-small cell lung carcinoma (NSCLC) or immune cells, such as T cells and DCs in the TIME (8). In a glioma mouse model, miR-138 treatment is positively associated with median survival time and negatively correlated with tumor regression (68). While some other miRNAs participate in tumor development. For example, the elevated expression of miR-1269 promotes the formation and progression of gastric cancer and suppresses cell apoptosis by modifying the AKT and Bax/Bcl-2 signaling pathways (69). The overexpression of miR-9 has been confirmed in glioma cells and reported to significantly improve their migration and invasion by targeting COL18A1, THBS2, PTCH1, and PHD3 (70). In cancer immunity, the function of immune cells can also be suppressed by miRNAs (71).

Moreover, emerging evidence has shown that lncRNAs have multiple functions in regulation of cell proliferation, migration, invasion, and apoptosis in cancer progression (72–74). Additionally, lncRNAs may be pivotal regulators of TIME remodeling *via* several mechanisms, including the induction of Treg cells, inhibition of recruitment of macrophages, activation-induced cell death (ACID) of T lymphocytes, and the activation of Ca^2+^-triggered signaling (75–78).

## Reprograming of Immune Cells in Time

**Figure 2:**
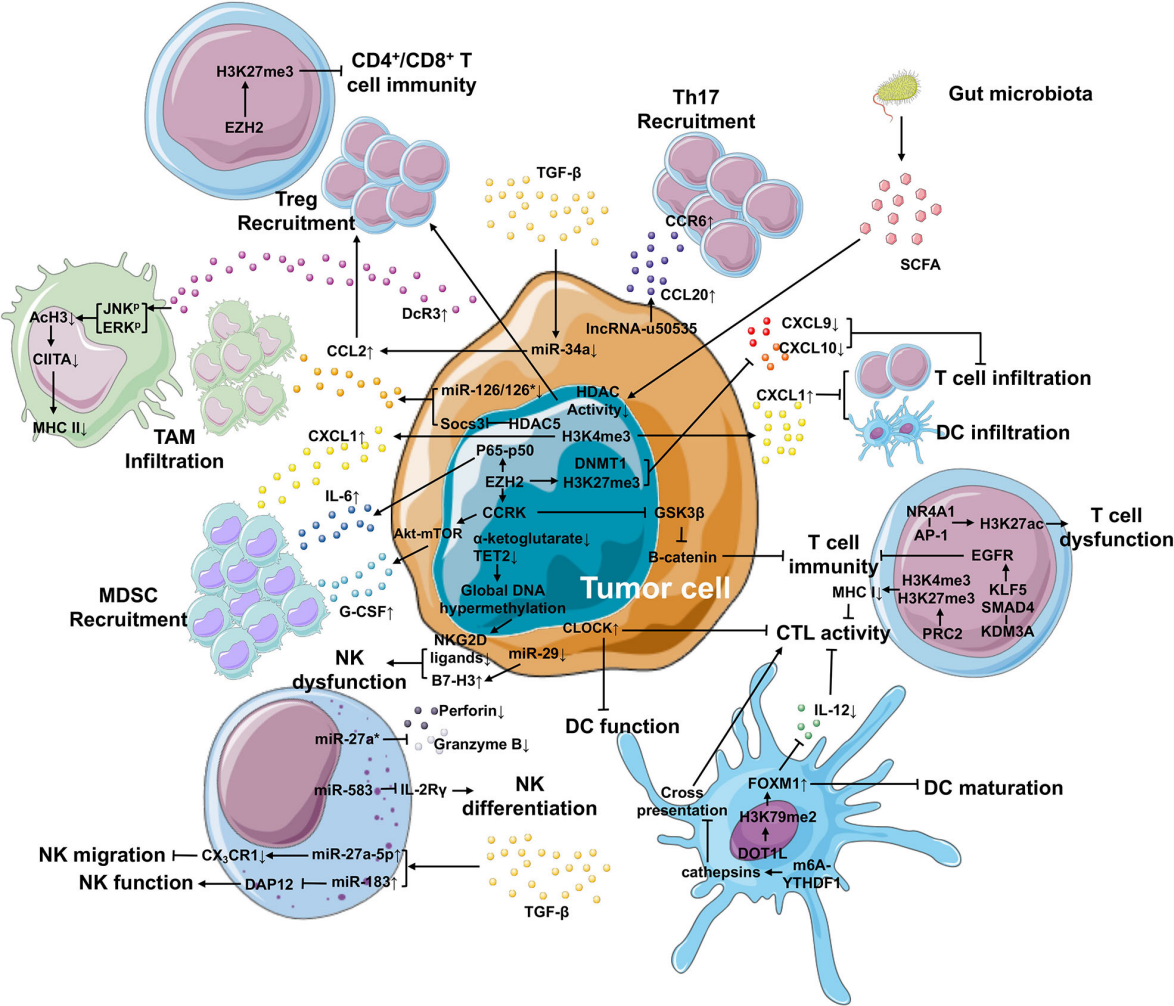
Epigenetic mechanisms in the TIME that contributes to cancer development. Within the TIME, epigenetic regulation plays an important role in generating immunosuppressive environment and facilitating tumor differentiation. In tumor cells, epigenetic regulation is involved in the upregulation of IL-6 and G-CSF and the downregulation of CXCL9 and CXCL10 *via* EZH2, as well as the elevated expression of CXCL1 secreted by tumor cells *via* H3K4me3, leading to improved MDSC recruitment and repressed T cell or DC infiltration, respectively. The expression of CCL2 (responsible for the recruitment of TAM and Treg cells) and CCL20 (responsible for Th17 recruitment) is enhanced by miR-126/126* or miR-34a and lncRNA-u50535, respectively. Furthermore, tumor cells can suppress the function of macrophage-, NK cell-, DC- and T cell-mediated immunity through other epigenetic mechanisms. In the TIME, high TGF-β levels can be produced by not only tumor cells, but also other cell types. TGF-β can regulate the expression of miRNAs in tumor cells and NK cells, suppressing NK migration and function and Treg recruitment. What’s more, the gut microbiota releases SCFA that inhibits the activity of HDACs, further improving the recruitment of Treg cells.

One of the biggest obstacles to cancer therapy is tumor escape from the host immune system. Tumor cells tend to modify the microenvironment around themselves by recruiting and educating immune cells, thereby forming an immunosuppressive area termed as TIME. Immune cells, including innate immune cells and T cells, support tumor expansion *via* various mechanisms, and the critical role of the epigenetic reprograming of these immune cells has been revealed (**Figure 2**). A multi-platform genome-wide dataset of various types of sarcoma demonstrated the correlation between epigenomic alterations and the infiltration of immune cells into the TIME (79).

### Innate Immune Cells

Macrophages are a type of white blood cells of the innate immune system that engulf and digest non-self substrates such as cancer cells in a process called phagocytosis. They have also been shown to contribute to tumor growth and progression after epigenetic modification into TAMs, the major infiltrating leukocytes in most malignant tumors. Research groups from the MD Anderson Cancer Center have performed gain-of-function screening of epigenetic regulators in an inducible Kras^G12D^ p53 null pancreatic ductal adenocarcinoma (PDAC) mouse model and identified that HDAC5 mediates the upregulated expression of chemokine CCL2 by repressing Socs3, resulting in the recruitment of TAMs, which subsequently enables KRAS*-independent tumor growth (80). CCL2 expression is regulated by miR-126/126* in breast cancer cells. Downregulated miR-126/126* by promoter methylation of their host gene *Egfl7* mediates CCL2 upregulation (81). Finally, elevated CCL2 recruit macrophages to promote breast cancer metastasis. MHC II molecules on the surface of macrophages mediate antigen presentation, which is important for the induction of adaptive immune responses. In patients with pancreatic cancer, ERK and JNK induce histone deacetylation at the promoter region of the class II transactivator (CIITA), leading to decoy receptor (DcR3)-mediated downregulation of MHC II expression (82). The loss of MHC II expression impairs the antigen presentation, resulting in TAM-induced immunosuppression (82). The differentiation and polarization of macrophages can also be modulated by the enhancer of zeste homolog 2 (EZH2) (83), a histone methyltransferase and the catalytic subunit of polycomb repressive complex 2 (PRC2), indicating that EZH2 is involved in the reshaping of TIME.

MDSCs (CD11b^+^Gr1^+^) are a heterogeneous group of immune cells from the myeloid lineage and possess strong immunosuppressive activities in cancer. In breast cancer patients, MDSC levels in the blood are approximately 10-fold higher than healthy individuals (84). Their expansion into the TIME is negatively correlated with poor survival rates due to inhibited CD8^+^ T cell proliferation in hepatocellular carcinoma (HCC) (85). In the same study, upregulated EZH2 interacts with the phosphorylated NF-κB subunit p65, and the EZH2-NF-κB complex binds to the *IL-6* promoter to enhance the expression of IL-6, thereby subsequently inducing MDSC recruitment to the TIME (85). In another study, the Akt-mTOR signaling pathway has been shown to trigger the recruitment of MDSCs to promote tumor initiation (86). And Akt phosphorylation can be mediated by cell cycle-related kinase (CCRK), whose expression can be regulated by EZH2 (87). These findings suggest that MDSCs can be recruited through distinct mechanisms associated with epigenetic modifications, especially those mediated by EZH2.

DCs are professional antigen-presenting cells (APCs) and act as messengers between the innate and adaptive immune systems. However, their antigen-presenting capacity is abolished in many solid tumors owing to their immature state and low levels of IL-12 production. Mechanistically, forkhead box M1 (FOXM1) expression is enhanced by H3K79me2 that is present in both tumor cells and DCs, which causes abnormal maturation phenotypes of DCs and decreased production of IL-12 in tumor-bearing mice with pancreatic and colon cancers (47). Furthermore, H3K79 is methylated by DOT1-like histone lysine methyltransferase (DOT1L) and the inhibition of DOT1L not only decreased H3K79me2, but also downregulated FOXM1 expression and reversed the immunosuppressive state (47). FOXM1 is reported to be associated with cancer proliferation, angiogenesis, EMT, migration, metastasis, and stemness in many types of cancer (88). A recent study has revealed that the RNA N6-methyladenosine (m^6^A) modification is correlated with TIME infiltration in gastric cancer (89). In DCs, the m^6^A modification mediated by RNA methyltransferase Mettl3 in the transcripts of CD40, CD80, and TLR4 signaling adaptor Tirap promotes the activation and function of DCs and DC-based T cell response (90). Han et al. have reported that the binding of YTH N6-methyladenosine RNA binding protein 1 (YTHDF1) to the transcripts encoding lysosomal proteases modified by m^6^A methylation improved the translational efficiency of lysosomal cathepsins in DCs, whereas the suppression of cathepsins in DCs significantly strengthened its ability to cross-present tumor antigens, which in turn enhanced the tumor infiltrating CD8^+^ T cell antitumor response (91). Through screening of known epigenetic regulators, the circadian locomotor output cycles kaput (CLOCK), a circadian regulator possessing potential histone acetyltransferase activity, has been shown to have a negative correlation with the function of CD8^+^ activated T cells and DCs in glioblastoma (GBM) (92). However, further studies are needed to elucidate the epigenetic regulation mechanism of CLOCK in TIME.

NK cells are cytotoxic lymphocytes critical to the innate immune system. Their role is analogous to that of cytotoxic T lymphocytes (CTLs), which recognize target cells such as cancer cells upon the expression of non-self HLA antigens. NKG2D ligands (ULBP1 and ULBP3) on tumor cells are downregulated *via* DNA methylation, resulting in the escape of IDH1 and IDH2 mutant gliomas from NK cells (93). IDH1 and IDH2 mutations cause global DNA hypermethylation because of decreased α-ketoglutarate levels and TET2 function in many cancer types, including acute myelogenous leukemia (AML) (93). The cytotoxicity of NK cells is also regulated by miRNAs. It is well known that B7-H3, a surface glycoprotein, exerts inhibitory effects on NK cells, which abolishes the anti-tumor activity of these cells (94). The downregulation of miR-29 expression in cancer contributes to the B7-H3 upregulation, leading to NK cell dysfunction and tumor immune escape (95, 96). Perforin (Prf1) and granzyme B (GzmB) are key cytotoxic effectors that kill cancer cells for NKs. However, miR-27a* reverses the cytotoxicity of NK cells by silencing Prf1 and GzmB expression (97). Because Prf1 and GzmB are the functional effectors of CTLs, the cytotoxic capacity of CTLs may also be inhibited by miR-27a*. Using a genome-wide mRNA and miRNA database, Yun et al. identified that miR-583 targets the 3’-UTR of the IL2 receptor gamma (IL2Rγ) and acts as a negative regulator of NK cell differentiation (98). The activity of NK cells is strongly repressed by TGF-β, an immunomodulatory cytokine that is released in the TIME. TGF-β induces the overexpression of miR-27a-5p, which targets 3’-UTR of the chemokine receptor CX_3_CR1 expressed in several immune cells, resulting in the suppression of the migration ability of NK cells (99). Another TGF-β-induced miRNA is miRNA-183. The miR-183 binds and suppresses the DNAX activating protein 12 kDa (DAP12), an adaptor protein critical for NK cells, to inhibit NK cell function, thus creating an immunosuppressive TIME (100).

### T Cells

The key effector cells for tumor eradication are the CD8+ cytotoxic T cells because they directly recognize and kill cells displaying foreign antigens through binding MHC I molecules. The loss of MHC I expression in tumor cells abolishes antigen presentation, thereby contributing to immune evasion. A genome-wide CRISPR/Cas9 screen was performed and identified that PRC2, a complex with histone methyltransferase activity, silences the expression of MHC I *via* bivalent H3K4me3 and H3K27me3 modifications and inhibits the anti-tumor immunity mediated by T cells (101). Simultaneously, the existence of bivalent H3K4me3 and H3K27me3 at the MHC I promoter region in a range of human MHC I-deficient cancers was detected (101). Thus, targeting bivalent H3K4me3 and H3K27me3 may be one of the potent therapeutic approaches in cancer treatment. Another *in vivo* CRISPR screen in a PDA mouse model identified that KDM3A potentially blocks T cell-mediated immune response *via* regulating the expression of epidermal growth factor receptor (EGFR) through the Krueppel-like factor 5 (KLF5) and SMAD family member 4 (SMAD4) (102), which makes KDM3A a potential target for cancer therapy.

ICI therapy for various cancers has revolutionized the standard of care and achieved significant clinical outcomes. Nevertheless, only a limited subset of patients harbors positive feedback after ICI treatment (103). The main reason for this is that the expression of immune checkpoint molecules/ligands is always regulated by epigenetic alterations, including DNA methylation, histone modification, and ncRNAs. Epigenetic regulation of immune checkpoint proteins on T cells can lead to an immunosuppressive TIME through the following effects: less responsive T cells, increased Treg cells, MDSC recruitment, and impaired release of effector cytokines (104). The Cancer Genome Atlas (TCGA) Level 1 methylation data from 30 solid tumor types have revealed that hypermethylated costimulatory genes and hypomethylated immune checkpoint genes are negatively associated with functional T cell recruitment to the TIME (105). To promote the therapeutic efficacy of ICI treatment, methods that can be used to restimulate the expression of immune checkpoint proteins and costimulatory molecules are one of the solutions in cancer therapy.

As mentioned above, EZH2 epigenetically upregulates the expression of CCRK, and CCRK inactivates GSK3β *via* phosphorylation, thus further activating β-catenin in HCC cells (87, 106). In addition, β-catenin signaling in melanoma samples is correlated with the absence of a T cell gene expression signature (107). These results suggest a relationship between EZH2 and CD8^+^ T cell infiltration within the TIME in melanoma. Regarding to T cell infiltration, CXCL1 overexpression in PDA tumors can diminish the number of infiltrated T cells (108). In this study, a library of congenic cell clones from KPCY tumors was established, and the immune microenvironment was analyzed. In brief, they found that H3K4me3 modification at the *Cxcl1* promoter enhances the expression of CXCL1 in PDA tumor cell clones, leading to low infiltration of T cells and DCs, and the recruitment of MDSCs, which shapes the TIME and influences the outcome of immunotherapy (108). Effector T-cell trafficking to the TIME is mediated by T helper 1 (T_H_1)-type chemokines CXCL9 and CXCL10. Whereas, in a human ovarian cancer model, H3K27me3 induced by EZH2 and DNA methylation catalyzed by DNMT1 at their promoter regions repress the expression of CXCL9 and CXCL10 in tumor cells (109). Furthermore, the expression of EZH2 and DNMT1 in tumors is negatively correlated with CD8^+^ T cell infiltration within the TIME, as well as patient prognosis (109). Therefore, EZH2 can serves as a cancer therapeutic target. Infiltrated T cells may be dysfunctional because of different mechanisms, which may include nuclear receptor subfamily 4 group A member 1 (NR4A1) regulation. NR4A1 is highly expressed in tolerant T cells and can bind to activator protein 1 (AP-1) to promote H3K27ac, which leads to the activation of tolerance-related genes (110).

In the healthy state, Treg cells play a pivotal role in maintaining host immune homeostasis. However, in HCC tumors, TGF-β stimulation leads to the low expression of miR-34a, upregulates CCL2 and finally recruits more Treg cells to the TIME (111). EZH2, an important methyltransferase, is considered as a potent therapeutic target in many cancers. The distinct expression level of EZH2 in Treg cells depends on their locations. Particularly, Treg cells in tumor tissues specifically express high levels of EZH2 and its histone modification H3K27me3 compared with those in non-lymphoid tissues, resulting in tumor tolerance (112). In addition, the EZH2 and H3K27me3 levels are increased only in Treg cells when compared to CD4^+^Foxp3^-^ T cells in tumor tissues (112). Targeting EZH2 in Treg cells remodels the TIME by improving recruitment and function of CD4^+^ and CD8^+^ effector T cells that guide antitumor immunity (112). The presence of Th17 cells (a group of CD4^+^ T cells characterized by RORγ expression and IL-17 production) in the TIME is correlated with poor prognosis in colorectal cancer patients. Th17 cells can be recruited to the TIME *via* the CCR6-CCL20 pathway in cervical cancer due to upregulated CCL20 in tumor tissues and high expression of CCR6 on Th17 cells aggregated within tumor tissues (113). It is a possible that Th17 cells are recruited into the TIME *via* the CCR6-CCL20 axis, thereby contributing to the lncRNA u50535-mediated tumor growth and metastasis of CRC (114, 115). In addition to CD8^+^ T cells, how to regulate the Treg cells and Th17 cells in TIME is also a viable option to improve the clinical outcome of cancer therapy.

In recent years, the gut microbiota has received increasing interest as they have been revealed ton interact with many human diseases, including cancer not only limited to colorectal cancer but also extraintestinal tumors (116). The gut microbiota can affect the DNA methylation patterns, chromatin structure, and miRNA activity to maintain the host immune system and homeostasis through the microbes themselves or metabolites (117–119). Butyrate, a short chain fatty acid (SCFA) derived by gut microorganisms, inhibits HDAC activities and induces an abundance of Treg cells, leading to tumor suppression in colitis-associated cancer (CAC), a major subset of CRC (120, 121). However, the relationship between HDAC inhibition and Treg cell recruitment in CRC needs to be clarified. Cancer immunotherapy requires microbiota-derived signals because the function of DCs for priming CD8^+^ T cells is controlled by the gut microbiota through H3K4me3, which activates genes related to immune responses (122, 123). There is limited evidence illustrating the mechanism of epigenetic modification between gut microbiota and TIME, which makes this area being an interesting field for researchers to investigate.

## Epigenetics in Intratumoral Heterogeneity

**Figure 3:**
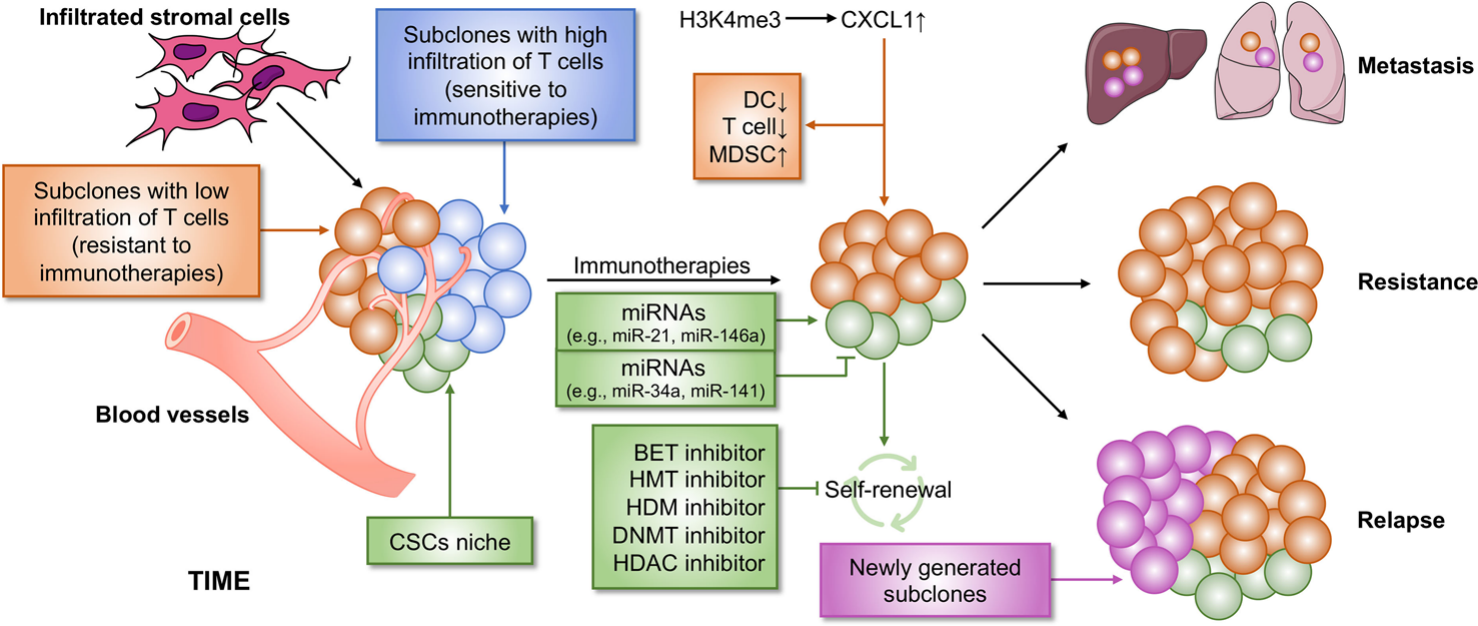
Epigenetic regulation of ITH that contributes to immunotherapy resistance. Cancer is a heterogeneous disease with a complicated TIME, leading to immunotherapy failure. Two main reasons contribute to immunotherapy resistance. First, within the TIME, there are some subpopulations of tumor that are non-responsive to immunotherapy, which causes resistance to immunotherapy. Second, CSCs cause poor clinical outcome in immunotherapy. CSCs may be not completely eliminated by immunotherapy, which subsequently lead to tumor relapse and metastasis. Both the two processes can be regulated by epigenetics, suggesting that a combination of immunotherapy and epi-drugs may be an effective strategy for cancer therapy. BET, bromodomain and extra-terminal domain; HMT, histone methyltransferase; HDM, histone demethylase; DNMT, DNA methyltransferase; HDAC, histone deacetylase.

ITH is termed as subpopulations of cancer cells with different phenotypes and molecular features within a tumor and also contains heterogeneity of the TIME, resulting in tumor metastasis, drug resistance and tumor relapse (**Figure 3**). Cancer stem cells (CSCs), a small population of stem-like cancer cells within the TIME, are one of the two major frameworks for interpreting the causes of ITH (22). Accumulating evidence suggests that CSCs represent a heterogeneous population of cells that can be regulated by epigenetics, possessing tumorigenicity and metastasis. In breast cancer, the MLL4-mediated H3K4me2 and the CBP/p300-c-Myc complex-mediated H3ac contribute to self-renewal of CSCs by regulating the expression of epithelial–mesenchymal transition (EMT) regulators, such as SNAIL, ZEB1, and ZEB2, in the absence of KDM6A (124). KDM6A (also known as UTX), a component of the MLL complex, recruits LSD1, HDAC1, and DNMTs to form a complex that inhibits H3K4me2 and H3ac, and enhances DNA methylation at the promoter regions of SNAIL, ZEB1, and ZEB2, thereby resulting in abolished CSC self-renewal, tumor proliferation, and migration (124). However, the role of KDM6A in breast cancer remains controversy, and whether KDM6A can serve as a therapeutic target needs to be further investigated. The expansion of CSCs is also promoted by TWIST1, whose expression is elevated by the CBP-mediated H3ac at the promoter, in which CBP degradation is repressed by MTDH, a protein always associated with tumor progression, metastasis, and drug resistance (125). Several epigenetic inhibitors were investigated to block self-renewal of CSCs, including DNMT, HDAC, histone methyltransferase (HMT), histone demethylase (HDM), and bromodomain and extra-terminal domain (BET) inhibitors (126, 127). Additionally, epigenetic regulators, miRNAs, have an ability in modifying CSC development. For example, both miR-34a and miR-141 inhibit prostate cancer stem cells and metastasis by targeting CD44, a CSC marker (128, 129).

CSCs are demonstrated to be involved in immune resistance by multiple lines of evidence in many cancer types and therefore contribute to immunosuppressive TIME. One of the main CSC regulators, c-Myc, that is commonly expressed in many human cancers, can upregulate the expression of immune checkpoint molecules CD47 and PD-L1 (130, 131). Non-autonomously, CSCs from many solid tumors have been proven to be able to release a majority of immunosuppressive factors or cytokines, such as VEGF, TGF-β, IL-4, IL-6, IL10, PD-1, and others, among which many can help recruit suppressive immune cells, including TAMs, Treg cells, and MDSCs, and impair CD8^+^ T cell function (132, 133). Collectively, CSCs play a pivotal role in the remodeling of TIME to establish an immunosuppressive environment. Multiple therapeutic methods targeting CSCs have sprung up like mushrooms, such as NK cells, CSC-based DC vaccine, CSC-based T cells (including CAR-T), and monoclonal antibodies (133). Overall, targeting CSC-based immunotherapies is a potential effective strategy for cancer treatment.

Another major framework for interpreting the causes of ITH is clonal evolution (22). The concept clonal evolution was proposed by Nowell in 1976 for the first time (134). Throughout the process of tumor development, clonal evolution preferably proceeds in a branching rather than in a linear manner, and this leads to clonal and (epi)genetic diversity in different subpopulations (22). Cancer therapeutic responses in clinical are largely determined by the evolution of resistant subpopulations and the changes in cellular phenotypes (135). Moreover, cancer immunotherapy is mainly dependent on the degree of functional infiltrated T cells, which positively correlates with clinical outcome. However, the number of infiltrated T cells is discriminated among different subclones originating from a single tumor tissue isolated from a PDA mouse model and associated with epigenetic regulation (108).

First, an autochthonous mouse model, including mutated *Kras* and *p53*, of PDA expressing the YFP lineage tag (KPCY) was established. Then, tumor was isolated from KPCY mice and experienced a limiting dilution to generate tumor cell clones. The data showed that TIME is diverse among separated clones, in which low T cell clones correlated with low DC infiltration and high MDSC recruitment. Tumors formed from clones with low T cell infiltration negatively correlated with immunotherapeutic responses, demonstrating that ITH could induce tumor relapse in patients responsive to immunotherapy. Mechanistically, CXCL1 was highly expressed in the tumor clones with low T cell infiltration due to the high levels of H3K4me3 enriched at the promoter region of the *Cxcl1* gene. G-CSF, responsible for MDSC recruitment, was also expressed at high levels in the T cell low tumor clones. However, the exact number of Treg cells was also higher in T cell high clones than in low clones, suggesting a correlation between Treg cells and immunotherapy response, which needs to be further explored. The inhibition of H3K4me3 might be a potential method for eliminating T cell low tumor clones and could be combined with immunotherapy to completely eliminate whole tumor in PDA patients.

## Clinical Trials

The antitumor efficacy of epi-drugs has been proved in preclinical experiments with elevated antitumor immunity. Many epi-drugs have been applied to clinical trials, and their ability to eradicate cancer has been investigated. Here, we discuss the recent results of clinical trials involved in epi-drugs (**Table 1**).

**Table 1:** Recent clinical trials.

Epigenetic inhibitors	Target	NCT number	Conditions	Status	Reference(s)
**DNMT inhibitors**					
SGI-110	DNMT1	NCT01696032	Ovarian cancer	Phase I	(136, 137)
SGI-110	DNMT1	NCT01896856	Previously treated metastatic colorectal cancer	Phase I/II	(138)
**HMT inhibitors**					
EPZ-5676	DOT1L	NCT01684150	Advanced hematologic malignancies	Phase I	(139)
GSK2816126	EZH2	NCT02082977	Advanced hematological and solid tumors	Phase I	(140)
CPI-1205	EZH2	NCT02395601	B-cell lymphoma	Phase I	(141)
Tazemetostat	EZH2	NCT01897571	Advanced solid tumors and B-cell lymphomas	Phase I/II	(142, 143)
**HDAC inhibitors**					
Panobinostat	pan-HDAC	NCT00878436	Recurrent prostate cancer after castration	Phase I/II	(144)
Vorinostat	pan-HDAC	NCT01422499	Relapsed solid tumor, lymphoma or leukemia	Phase I/II	(145)
Vorinostat	pan-HDAC	NCT00731731	Newly diagnosed glioblastoma multiforme	Phase I/II	(146)
**Combinations**					
Decitabine/Valproic acid/Retinoic acid	DNMT/HDAC	NCT00867672	Acute myeloid leukemia	Phase II	(147)
Romidepsin/5-azacitidine	HDAC/DNMT	NCT01998035	Relapsed/refractory lymphoid malignancies	Phase I/II	(148)
Romidepsin/5-azacitidine	HDAC/DNMT	NCT01537744	Advanced solid tumors	Phase I	(149)
CC-486/pembrolizumab	DNMT/PD-L1	NCT02546986	Advanced or metastatic non-small cell lung cancer	Phase II	(150)
Vorinostat/pembrolizumab	HDAC/PD-L1	NCT02638090	Stage IV non-small cell lung cancer	Phase I/II	(151)
Vorinostat/pembrolizumab	HDAC/PD-L1	NCT02538510	Recurrent squamous cell head and neck cancer or salivary gland cancer	Phase I/II	(152)

### DNMT Inhibitor

Guadecitabine (SGI-110), a next-generation DNMT inhibitor, is under investigation in clinical trials for its ability of resistance to degradation by cytidine deaminase, leading to a prolonged activity *in vivo*. It has been confirmed that SGI-110 is able to improve the expression of HLA class I molecule on melanoma cells and the number of CD8+ T cells and CD20+ B cells, which demonstrated that SGI-110 has promising immunomodulatory and antitumor capacity (153).

In a phase I clinical trial for PK/PD analysis, 20 patients with recurrent, platinum-resistant ovarian cancer were enrolled and administered with guadecitabine and carboplatin (136). The first six patients treated with 45 mg/m^2^ of guadecitabine and carboplatin AUC5 reported neutropenia and thrombocytopenia, while the remaining 14 patients who were treated with 30 mg/m^2^ of guadecitabine and carboplatin AUC4 reported no such toxicity. Furthermore, three patients had a partial response (PR) and 15% clinical benefit rate (CBR), and six patients performed stable disease (SD) for more than 3 months with 45% CBR. Additionally, a CA-125 reduction of at least 50% was observed in 5/15 evaluable patients. In summary, this phase I clinical trial demonstrated the efficacy and safety of guadecitabine and carboplatin combination therapy in a platinum-resistant ovarian cancer cohort, supporting a completed Phase II trial (137).

Another phase I trial on guadecitabine was conducted in 22 previously irinotecan-treated patients with metastatic colorectal cancer (mCRC) (138). They were treated across four doses: guadecitabine 30 mg/m^2^ with or without growth factor support (GFS) and guadecitabine 45 mg/m^2^ with or without GFS. Each patient received 125 mg/m^2^ irinotecan at days 8 and 15. At the endpoint of this trial, the median overall survival (OS) was 10.7 months, and 17 patients were evaluable, among which, 12 had SD as the best response and five had PD. Using LINE-1 analysis, global DNA demethylation in tumors was found to be decreased as expected. What’s more, guadecitabine 45 mg/m^2^ and irinotecan 125 mg/m^2^ with GFS showed the least severe side effects in mCRC patients. These findings provide a theoretical basis for a subsequent randomized phase II trial. In elderly non-fit patients with AML, the combination of retinoic acid and decitabine led to a higher remission rate and increased median overall survival, without additional toxicity (147).

### HMT Inhibitor

Pinometostat (EPZ-5676) is a first-in-class inhibitor of DOT1L, which plays a central role in Th cell lineage commitment and stability, and has been evaluated as a single agent for the treatment of adult patients with advanced acute leukemia, especially those with mixed-lineage leukemia gene rearrangements (*MLL-r*) leukemia. After treatment, only two patients experienced complete remission at 54 mg/m^2^ per day, demonstrating the clinical benefit of EPZ-5676 for *MLL-r* patients (139).

EZH2 is another attractive target for anti-cancer therapy because of its ability in promoting the division and proliferation of cancerous cells and role in regulating immune cells in TIME, including T cells, NK cells, DCs and macrophages (154). Reprograming the TIME by targeting EZH2 is a viable area of cancer research (112, 155). At present, there are three different EZH2 inhibitors, namely tazemetostat, GSK2816126, and CPI-1205, which have been investigated in phase I clinical trials. After treatment with tazemetostat, the most commonly reported adverse event (AE) was asthenia (33%) in 64 patients (21 with B-cell non-Hodgkin lymphoma and 43 with advanced solid tumors) (142). Among these, no treatment-related deaths occurred, and durable objective response rates were 38 and 5% in patients with B-cell non-Hodgkin lymphoma and solid tumors, respectively (142). GSK2816126, a highly selective inhibitor of EZH2, was applied for the treatment of 41 patients with solid tumors or B cell lymphoma (140). In this trial, 12 (32%) patients had a severe AE, and fatigue (53.7%) and nausea (48.8) were the most common toxicity (140). PK/PD results showed that the half-life of GSK2816126 was approximately 27 h and its maximum tolerated dose (MTD) was 2,400 mg (140). Finally, 14 (34%) patients experienced the best response of SD and 21 (51%) patients had progressive disease (140). CPI-1205, the third selective EZH2 inhibitor, was orally administered twice a day in 32 patients with B-cell lymphomas (141). CPI-1205 had the shortest half-life (~3 h) among the mentioned three EZH2 inhibitors, but induced grade 2 or lower drug-related AEs (141). Among patients, only one achieved a complete response (CR) and five patients had SD (141). Based on these findings, ongoing research needs to be conducted using CPI-1205 in combination in solid tumors (141).

### HDAC Inhibitor

HDAC inhibitors have been proved to be able to alter the secretion level of cytokines and chemokines, favoring a Th1 immune response in cancer therapy (156). Panobinostat, a pan-HDAC inhibitor, has been approved by FDA for use in multiple myeloma patients in 2015 and able to improve NK cell-mediated tumor eradication (156, 157). In a phase I/II clinical trial, panobinostat was combined with bicalutamide to treat patients with castration-resistant prostate cancer (CRPC) and restore the resistance to bicalutamide in CRPC patients (n = 64; Phase I: 9; Phase II: 55) (144). In the phase II trial, panobinostat at 40 mg p.o. triweekly was selected as the highest oral dose based on the Phase I trial (144). The median time to PSA progression was 9.4 and 6.3 weeks for the A and B arms, respectively (144). The most common AE for the two arms was fatigue (55 and 65%, respectively), and the toxicity of panobinostat was tolerable with dose reductions (144). Overall, panobinostat, together with bicalutamide, increased rPFS in CRPC patients and reduced androgen receptor-mediated resistance to bicalutamide (144).

HDAC inhibitors can also be combined with DNMT inhibitors for the treatment of lymphomas, AML, and solid tumors. In a phase I study, 5-azacytidine (a DNMT inhibitor) and romidepsin (a HDAC inhibitor) were combined for the treatment of patients with peripheral T-cell lymphoma (PTCL) (148). This combination therapy was well-tolerated in lymphoid malignancy patients and produced a better overall response rate (73%) and complete response rate (55%) in patients with PTCL than in those with non-T-cell lymphoma (148). Combined with the DNMT inhibitor CC-486, romidepsin was investigated in another phase I clinical trial, in which 18 patients with advanced solid tumors were enrolled (149). Although the combination of CC-486 and romidepsin was tolerable, the antitumor effect was not significant (149). Another HDAC inhibitor vorinostat was investigated in two Phase I/II clinical trials as a single agent or in combination therapy (145, 146).

### Combination Therapy With ICI

Most patients exhibited no or partial response to ICI therapy, which is attributed to several factors, including tumor mutational burden (TMB), TIME and tumor immune evasion (9). Owing to the function of epigenetic regulation in malignancies, the combination of epi-drugs and ICI therapy may be open a new gate for cancer therapy, especially DNMT inhibitor and HDAC inhibitor (158).

A randomized phase II study was conducted to compare the treatment efficacy and safety of pembrolizumab (PD-L1 mono-antibody) plus CC-486 or placebo in NSCLC patients previously treated with platinum (150). Unfortunately, no improved PFS was shown between pembrolizumab + CC-486 and pembrolizumab + placebo arms. The treatment feasibility might be influenced by AEs, particularly gastrointestinal, thus resulting in non-comparable median OS (11.9 months vs. not estimable) (150).

Two clinical trials, a phase I/Ib and a Phase II, were performed using pembrolizumab and vorinostat combination therapy in patients with NSCLC, and head and neck (HN) and salivary gland cancer (SGC), respectively. The phase I/Ib study demonstrated that pembrolizumab (200 mg) plus vorinostat (400 mg) were the recommended dose which was well tolerated (151). Among the enrolled 33 patients, 30 were evaluable for response: four (13%) had partial response; 16 (53%) had SD; and 10 (33%) had progressive disease (151). In the ICI-pretreated cohort, CD8^+^ T cell presence in the tumor stromal area was correlated with treatment benefit (151). While MDSCs showed no such association. Another combination therapy involving pembrolizumab and vorinostat was investigated in a phase II trial conducted in 25 HN and 25 SGC patients (152). The toxicities of this combination therapy were more severe than those of pembrolizumab alone reported elsewhere. The median OS and median PFS were 12.6 and 4.5 months and 14 and 6.9 months in the HN and SGC cohorts, respectively. Beneficial responses in SGC were reportedly fewer than those in HN when treated with pembrolizumab and vorinostat, possibly due to the low expression of PD-L1 on SGC.

## Conclusion

Epigenetic regulation (DNA methylation, histone modification, and ncRNAs) plays a controversial role in cancer initiation and progression, especially in the modification of TIME. Epigenetics-related drugs approved by FDA are proved to be sufficient for cancer therapy, suggesting that targeting epigenetic pathway is a promising strategy for cancer treatment. This strategy can not only induce anti-proliferation of tumor cells, but also shift the TIME from cold to hot. Moreover, the gut microbiota-mediated epigenetic regulation can also influence tumor cells and the host immune system; however, the mechanism by which the microbiota epigenetically shape TIME needs to be further investigated. Another interesting area of research is the epigenetic regulation of B cell function in tumor development. Because of ITH, therapies targeting each tumor clone and CSCs represent new directions for cancer treatment.

Both pre-clinical and clinical studies have confirmed the antitumor effect of epi-drugs. However, a single epi-drug had not achieved much positive feedback in clinical trials, demonstrating that epi-drugs should be employed in combination with other cancer therapeutic approaches, including chemotherapy, radiotherapy, and immunotherapy, particularly ICI therapy. Due to the toxicity of epi-drugs, ongoing research should focus on how to decrease their side effects. ncRNAs are well-known group of factors that regulate tumor development. Thus, combination of ncRNA-related drugs and immunotherapy may be another potential strategy for cancer treatment in clinical trials.

## Author Contributions

YY and YW wrote the manuscript. YW critically revised the manuscript. All authors contributed to the article and approved the submitted version.

## Funding

This work was supported by the National Natural Science Foundation of China (No. 41931291, No. 81773017 to YW), the Chinese Academy of Medical Science Innovation Fund for Medical Sciences (CIFMS; No. 2019-I2M-1-003), and the Non-profit Central Research Institute Fund of Chinese Academy of Medical Sciences (2019PT310027).

## Conflict of Interest

The authors declare that the research was conducted in the absence of any commercial or financial relationships that could be construed as a potential conflict of interest.
